# Dynamically Accumulating Homologous Recombination Deficiency Score Served as an Important Prognosis Factor in High-Grade Serous Ovarian Cancer

**DOI:** 10.3389/fmolb.2021.762741

**Published:** 2021-11-19

**Authors:** Rongjia Su, Yuan Liu, Xiaomei Wu, Jiangdong Xiang, Xiaowei Xi

**Affiliations:** ^1^ Department of Obstetrics and Gynecology, Shanghai General Hospital, Shanghai Jiaotong University School of Medicine, Shanghai, China; ^2^ Reproductive Medicine Center, Department of Obstetrics and Gynecology, Shanghai General Hospital, Shanghai Jiaotong University School of Medicine, Shanghai, China

**Keywords:** BRCA, homologous recombination deficiency, ovarian cancer, PARP inhibitors, WGCNA, overall survival

## Abstract

**Background:** The homologous recombination (HR) pathway defects in cancers induced abrogation of cell cycle checkpoints, resulting in the accumulation of DNA damage, mitotic catastrophe, and cell death. Cancers with BRCA1/2 loss and other accumulation of similar genomic scars resulting in HRD displayed increased sensitivity to chemotherapy. Our study aimed to explore HRD score genetic mechanisms and subsequent clinical outcomes in human cancers, especially ovarian cancer.

**Methods:** We analyzed TCGA data of HRD score in 33 cancer types and evaluated HRD score distribution and difference among tumor stages and between primary and recurrent tumor tissues. A weighted gene co-expression network analysis (WGCNA) was performed to identify highly correlated genes representing essential modules contributing to the HRD score and distinguish the hub genes and significant pathways. We verified HRD status predicting roles in patients’ overall survival (OS) with univariate and multivariate Cox regression analyses and built the predicting model for patient survival.

**Results:** We found that the HRD score increased with the rise in tumor stage, except for stage IV. The HRD score tended to grow up higher in recurrent tumor tissue than in their primary counterparts (*p* = 0.083). We constructed 15 co-expression modules with WGCNA, identified co-expressed genes and pathways impacting the HRD score, and concluded that the HRD score was tightly associated with tumor cells replication and proliferation. A combined HRD score ≥42 was associated with shorter OS in 33 cancer types (HR = 1.010, 95% CI: 1.008–1.011, *p* < 0.001). However, in ovarian cancer, which ranked the highest HRD score among other cancers, HRD ≥42 cohort was significantly associated with longer OS (HR = 0.99, 95% CI: 0.98–0.99, *p* < 0.0001). We also built a predicting model for 3 and 5 years survival in HGSC patients.

**Conclusion:** A quantitative HRD score representing the accumulated genomic scars was dynamically increasing in proliferating tumor cells since the HRD score was tightly correlated to tumor cell division and replication. We highlighted HRD score biomarker role in prognosis prediction of ovarian cancer.

## Introduction

Flawed DNA damage repair and genome instability are associated with additional susceptibility to cancer and are found significantly in most cancer types (Hoeijmakers, 2009). Studies showed that the homologous recombination (HR) pathway was altered in nearly 40% of cancers, for example, in ovarian and triple-negative breast cancers. High-grade serous ovarian cancer (HGSC) is the most common and aggressive type of epithelial ovarian cancer, with homologous recombination deficiency (HRD) and germline or somatic BRCA1/2 mutation being the high risk of oncogenesis (Daly et al., 2021). HR pathway malfunction included gene mutation of the pathway in tumor cells and displayed defects in several aspects of DNA repair (Turner et al., 2004; Silver and Livingston, 2012). Many anticancer agents act by creating DNA damage, which induces cell death or senescence if unrepaired. BRCA1/2-mutated ovarian cancer, breast cancer, and pancreatic cancer were found to be sensitive to inhibitors of PARP, a single-strand break repair protein, specifically a target therapy through “synthetic lethality.” Ccells with defects in the HR pathway cannot mend DNA breaks and replication forks and are particularly susceptible to DNA damage and cell death induced by poly(ADP-ribose) polymerase inhibitors (PARPis). There were still pending questions in clinical treatment though the application of PARPis, and the related tumor biomarker tests have been advocated widely.

Some clinical phenomena have attracted our interest. First, for the patients with germline BRCA1/2 mutation who were sensitive to PARPis, human somatic cells with theoretically subsequent BRCA1/2 mutation were not globally harmed by PARPis, which meant that somatic and tumor cells demonstrated varied sensitivity to PARPis. Second, myelosuppression was present in a largely varying degree of patients receiving PARPis. Some patients could continue PARPis maintenance for years, while others have to cease due to the severe hematological toxicities. Is there other potential mechanism explaining the different extents of PARPis side effects? Third, there are always patients with germline BRCA1/2 mutation that showed no response to PARPis and short treatment free interval. In these patients, PARPis were not able to attack BRCA-mutated cells including neoplastic and non-neoplastic cells. Fourth, in VELLA and PAOLA-1 clinical trials, frontline HGSC patients showed significantly prolonged progression-free survival (PFS) in HR-deficient population compared to HR-proficient population (Coleman et al., 2019; Ray-Coquard et al., 2019). However, NOVA and ARIEL3 clinical trials illustrated that platinum-sensitive recurrent HGSC patients showed general response to PARPis, regardless of gene test results (Coleman et al., 2017; Del Campo et al., 2019). Recurrent tumors without BRCA mutation also demonstrated elevated sensitivity to PARPis. Thus, we speculated that PARPi therapeutic effects possibly depended not on BRCA mutation condition but on other mechanisms.

These questions have shed light on the mechanism of HR pathway deficiency. First, a functional HR pathway has played an essential role in ensuring tumor cell survival after defective DNA damage. HR-deficient HGSCs are P53 ectopic-expressed and have invalidation of cell cycle checkpoints resulting in the accumulation of DNA damage, mitotic catastrophe, and cell death (Evers et al., 2010; Cancer Genome Atlas Research Network, 2011). HR-deficient tumors display large (>15 Mb) sub-chromosomal deletions and lodge allelic imbalance stretching to the telomeric end of the chromosomes with or without changes in the overall DNA copy number (Konstantinopoulos et al., 2015). Second, HR deficiency may occur in epithelial ovarian cancer *via* multiple mechanisms, including BRCA1/2 mutations. From TCGA data, approximately 50% of HGSCs have alterations in HR repair genes and were referred to as HRD. Notably, BRCA1/2 mutation only makes up half of the HRD population (Konstantinopoulos et al., 2015). Genetic and epigenetic changes in the HR pathway other than BRCA1/2 mutation could also lead to HR deficiency like BRCA1 promoter methylation, CDK12 mutation, RAD51C promoter methylation, Fanconi gene methylation, and core RAD gene mutation (Baldwin et al., 2000; Cancer Genome Atlas Research Network, 2011; Loveday et al., 2011). Third, one method to determine if a patient has HRD is to measure genomic instability as a downstream consequence of non-precision double-strand break (DSB) repair. It evaluated the genomic footprints caused by the loss of the HR function through single-nucleotide polymorphism (SNP) array data. In the myChoice CDx assay, HRD is evaluated by a genomic instability score (GIS), characteristic of defective DNA repair. The result is made according to the calculated percentage of loss of heterozygosity (LOH), telomeric allelic imbalance (TAI), and large-scale state transitions (LSTs) (Watkins et al., 2014). A combined HRD score was the unweighted numeric sum of the three component scores (Telli et al., 2016).

Here, we analyzed the data from The Cancer Genome Atlas (TCGA) to systematically analyze genetic mechanisms of HR deficiency and the resulting consequences in 33 cancer types. We provided later the HRD score alterations and the subsequent clinical outcomes in human cancers and tried to shed light on unresolved clinical problems. Our analysis underlined the role of HRD score in precise medical therapy of HGSC.

## Methods

### Data Download

The datasets including 1. signatures–HRD score and genome-wide DNA damage footprint; 2. phenotype-curated clinical data; and 3. gene expression RNAseq-TOIL RSEM TPM were downloaded from https://xenabrowser.net/. It included 12,591 cancer cases across 33 types of cancer types with clinical information, and 10,647 cases with HRD score signature. After merging, 10,619 cases were extracted. After removing cases of less than 30 days of survival, 10,171 cases were exacted for the survival analysis of 33 cancer types. The clinical information is listed in Supplementary Material S1. Additionally, gene expression RNAseq of 10,535 cases was downloaded from TPM data (Transcripts PerKilobase Million) of the website mentioned before and from which 9,335 cases with the HRD score were extracted for the WGCNA analysis.

From cases with clinical information, 7,973 cases with both the HRD score and the AJCC pathologic tumor stage or clinical stage were extracted. Next, from the 7,973 cases, 342 cases with BRCA1/2 mutation referred to HR deficiency were picked for further analysis. From the 7,973 cases, 27 cases with paired primary and recurrent cancer information (Supplementary Material S2) were picked and displayed by ggplot2 and ggpubr R packages. HRD scores from the 27 cases were compared between primary and recurrent tumor tissues by a paired-samples *t* test.

Clinical information of 488 cases of primary HGSC were from the attachment of the article “Integrated Genomic Analyses of Ovarian Carcinoma,” specifically 2010-09-11380C-Table_S1.2. xlsx. 473 cases of primary HSCG with an HRD score were exacted. The detailed clinical information of 473 HGSC cases is listed in Table 1. After removing cases of survival days less than 30 days and one case with missing survival information, 459 cases were extracted for survival analysis. Next, 328 cases after removing cases with missing signatures were used for predicting the model.‬‬‬‬‬‬‬‬‬‬‬‬‬

**TABLE 1:** Clinical information of 473 high-grade serous ovarian adenocarcinoma cases.

	Deceased (N = 258)	Living (N = 210)	Overall (N = 473)
Age (years)			
Mean (SD)	61.6 (10.8)	58.7 (11.9)	60.2 (11.5)
Median [min, max]	60.7 [36.1, 84.7]	57.7 [30.5, 87.5]	59.1 [27.2, 87.5]
Missing	6 (2.3%)	3 (1.4%)	11 (2.3%)
Stage			
Stage II	7 (2.7%)	17 (8.1%)	24 (5.1%)
Stage III	202 (78.3%)	164 (78.1%)	369 (78.0%)
Stage IV	48 (18.6%)	28 (13.3%)	76 (16.1%)
Missing	1 (0.4%)	1 (0.5%)	4 (0.8%)
Grade			
G2	34 (13.2%)	23 (11.0%)	57 (12.1%)
G3	217 (84.1%)	184 (87.6%)	405 (85.6%)
Missing	7 (2.7%)	3 (1.4%)	11 (2.3%)
Tumor_residual (mm)			
>20	51 (19.8%)	32 (15.2%)	86 (18.2%)
1–10	135 (52.3%)	78 (37.1%)	213 (45.0%)
11–20	21 (8.1%)	9 (4.3%)	30 (6.3%)
No macroscopic disease	30 (11.6%)	59 (28.1%)	89 (18.8%)
Missing	21 (8.1%)	32 (15.2%)	55 (11.6%)
HRD_score			
Mean (SD)	43.9 (21.2)	49.0 (19.0)	46.2 (20.4)
Median [min, max]	41.0 [1.00, 99.0]	51.0 [1.00, 92.0]	44.0 [1.00, 99.0]

### Weighted Co-Expression Network Construction (WGCNA)

WGCNA is a systematic biological method used to build a gene co-expression networks to mine network modules closely associated with clinical traits (Langfelder and Horvath, 2008). In the present study, we used HRD component scores which are TAI, LST, and LOH and a combined score in every sample as target clinical traits. The top 25% genes with most median absolute division (MAD) used as a robust measure of variability were selected for WGCNA analysis. Next, an average linkage method was used for all pair-wise genes to construct a co-expression similarity matrix. The co-expression similarity matrix was then transformed into the adjacency matrix by choosing the soft-threshold parameter to ensure an unsigned scale-free network. Then we created a topological matrix using the topological overlap measure (TOM) (Li and Horvath, 2007). To classify genes with similar expression patterns into gene modules, the dynamic hybrid cut method, according to the TOM-based dissimilarity, was performed with the following major parameters: min module size of 30 and deep split of 3. Finally, a cutline of 0.25 was selected for module dendrogram. We merged some modules according to dissimilarity of estimated module eigengenes (MEs). MEs were defined as the first principal component of a given module and represented the gene expression patterns in a module (Langfelder and Horvath, 2007).

### Identification of Clinical Significant Modules and Hub Genes

The interesting modules were identified by calculating the relevance between clinical traits and MEs. The module highly correlated with target clinical trait was selected for further analysis. Hub genes were defined as highly interconnected within a module and have been shown functionally significant. Two approaches were used to identify hub genes in this study. First, potential hub genes were defined by module connectivity with Pearson’s correlation of module membership >0.8 and clinical characteristic relation with Pearson’s correlation of gene significance >0.2. Module membership (MM), which quantified how close a gene stayed with a given module, was referred to as the correlation between the ME and the gene expression profile. Gene significance (GS) was defined as the log10 transformation of the *p* value of each gene in the linear regression between gene expression and the clinical traits. The other way to search hub genes is to use the intramodule connectivity, which represents the relationship between genes within a specific module. Potential hub genes were defined by a connectivity degree ≥5 and the connectivity weight threshold set as 0.45 in the TOM-based co-expression network. After merging the results of the two aforementioned approaches, the modules of interest were constructed using Cytoscape 3.8.0 (Shannon et al., 2003). The genes with the higher MM, GS, and connectivity degree were more possibly considered as hub genes in the module of interest.

### Pathway Enrichment Analysis

To further explore the biological significance of hub genes, Gene Ontology (GO) and Kyoto Encyclopedia of Genes and Genomes (KEGG) pathway enrichment analysis for hub genes were conducted. Enriched pathways with a *p* value ≤0.05 were considered statistically significant.

### Survival Analysis

Univariate and multivariate Cox regression analyses were performed to evaluate the possible factors impacting OS using survival and survminer R packages. A log-rank test was applied to make the Kaplan–Meier curve. The HRD score ≥42 compared to the HRD score <42 was an important variable in the analysis. Other impacting factors included patient age, tumor stage, and tumor residual. A stepwise regression selection with bidirectional elimination was applied in the multivariate Cox regression analysis. A *p* value < 0.05 was considered to demonstrate a significantly difference. In addition, the log2 (HR), 95% CI, and statistical significance were calculated and illustrated using a forest plot through the forest plot R package. We established a prognostic nonogram on the basis of the results of the stepwise regression multivariate analysis. The performance of nomogram was displayed, followed by calibration plots. A nonogram was constructed by rms and regplot R packages. The calibration plots were performed by rms R package. A decision curve analysis was used to determine the clinical usefulness of the model and was generated by the stdca. R function.

## Results

### HRD Score Was Associated With Clinical Outcomes

**FIGURE 1:**
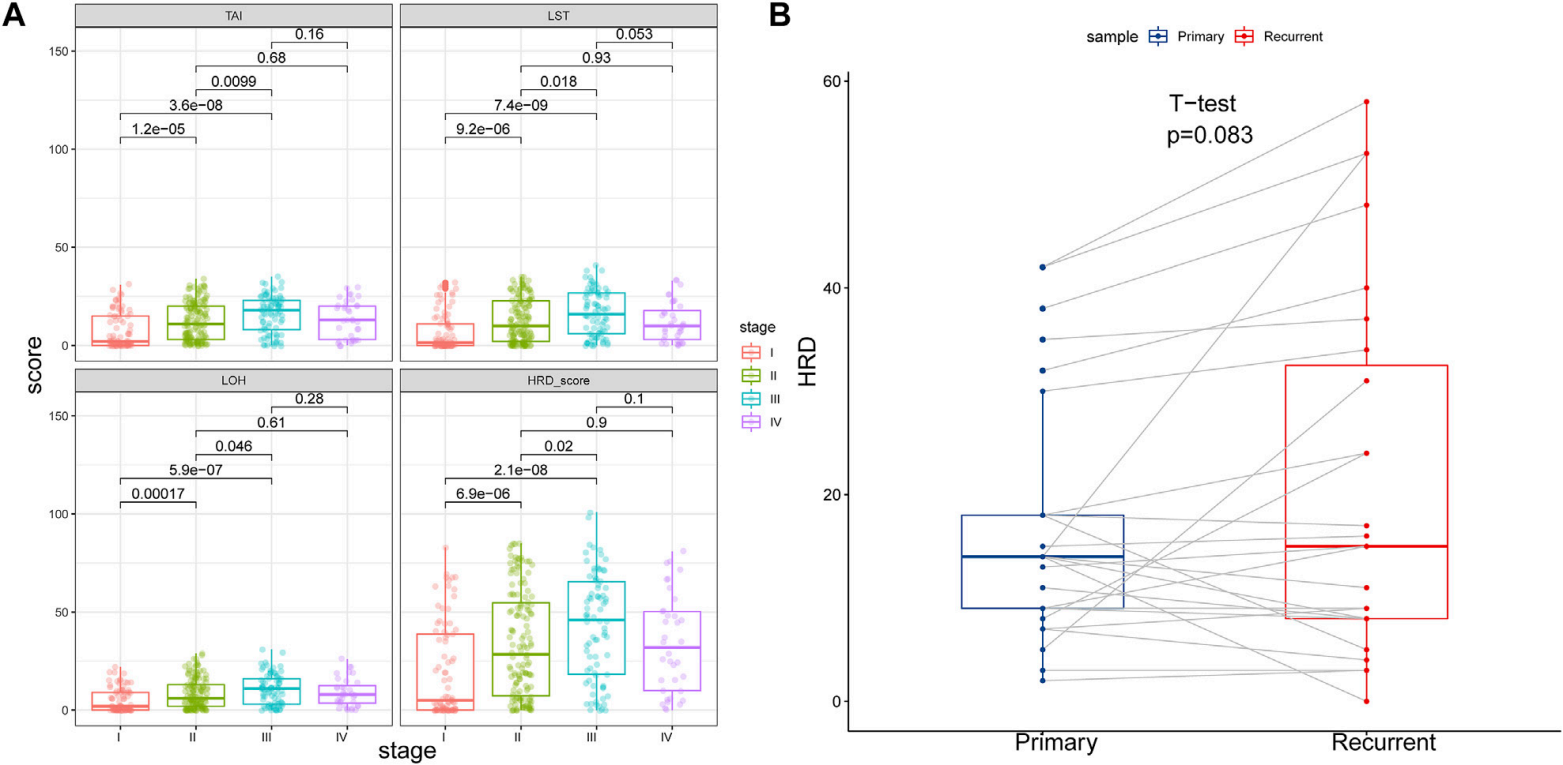
**(A)** HRD component scores and combined score in different tumor stage. HRD component scores and combined score were higher in stage II than in stage I, higher in stage III than in stage I, and higher in stage III than in stage II (*p* < 0.05). HRD component scores and combined score were similar between stage IV and stage III, and similar between stage IV and stage II (*p* > 0.05). **(B)** HRD combined score in primary and recurrent tumor samples. Recurrent tumor tissues showed similar HRD combined score with the primary counterpart (*p* = 0.083).

We computed the HRD scores and investigated their associations with clinical outcomes in altogether 33 cancer types. We investigated the component scores, which were TAI, LST, and LOH scores, and the combined HRD score. In 342 cancer cases with BRCA1/2 mutation, which was referred to as HR deficiency, a higher tumor stage was correlated with both higher HRD component scores and a higher HRD combined score, except for the comparison between stage IV and stage III and between stage IV and stage II (Figure 1A). Next, we retrieved and compared combined HRD scores between 27 pairs of primary and recurrent tumor specimens. According to Figure 1B, combined the HRD score (Supplementary Material S2) was slightly higher in recurrent than in primary tissue although the difference was not statistically significant (*p* = 0.083). These results suggested that the HRD score representing the accumulation of genomic scar was probably altered in the progression and recurrent of tumor development.

### Weighted Co-Expression Network Construction (WGCNA) for HRD Mechanism

**FIGURE 2:**
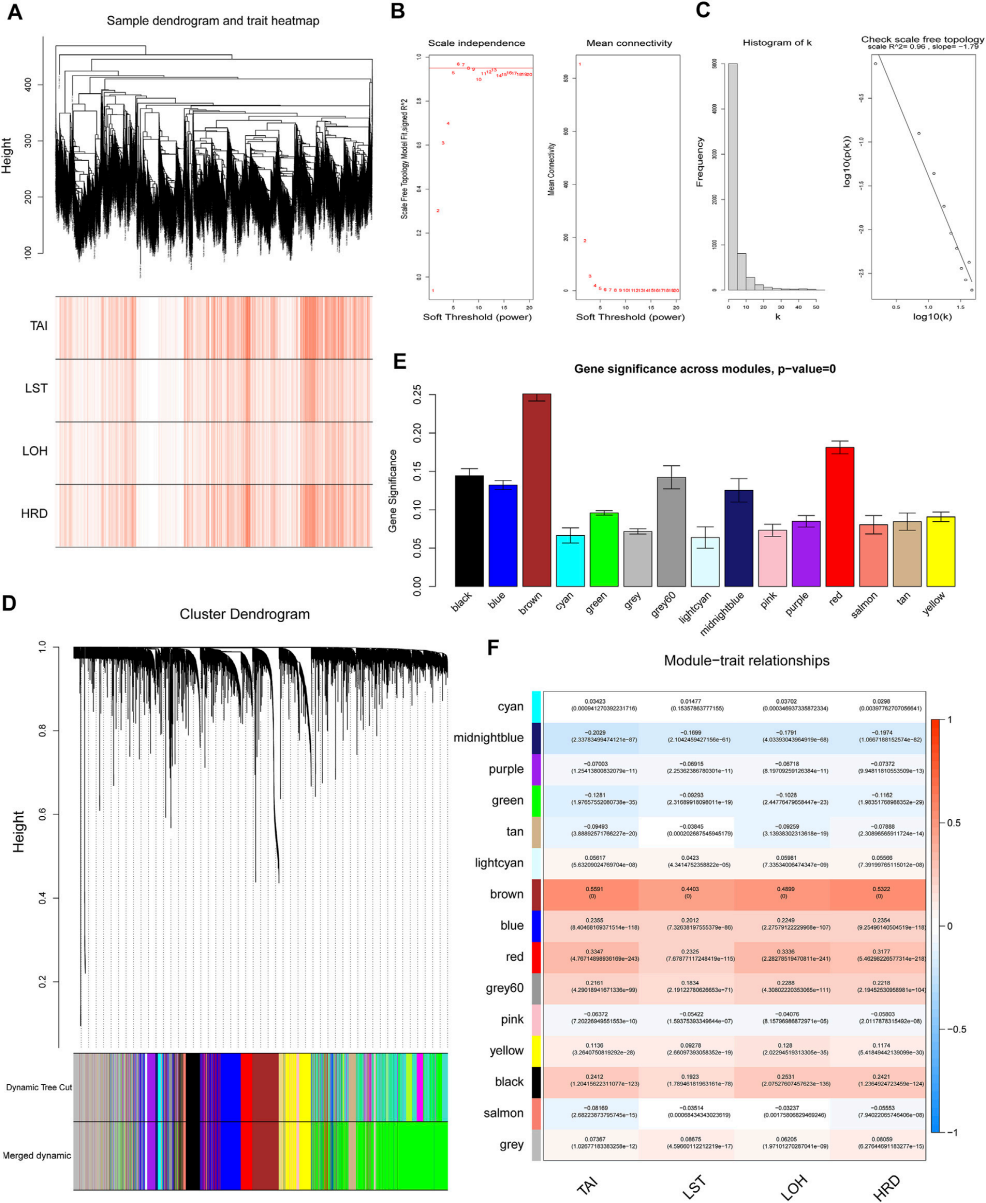
Weighted gene co-expression network analysis. **(A)** The sample dendrogram and trait heat map of 6,386 genes. **(B)** Analysis of scale-free topology for various soft-threshold powers (β), including the scale-free fit index and mean connectivity. **(C)** Scale-free network (scale-free *R* ^2^ = 0.96, slop = −1.79). **(D)** Dendrogram for the intersection of differentially expressed genes, clustered based on a dissimilarity measure, together with assigned module colors. **(E)** Gene significance across modules. **(F)** Analysis of module–trait relationships of endometriosis. Each row represents a module eigengene. Each column represents a trait. Groups indicate telomeric allelic imbalance (TAI), large-scale state transitions (LSTs), loss of heterozygosity (LOH), and HRD combined score.

**FIGURE 3:**
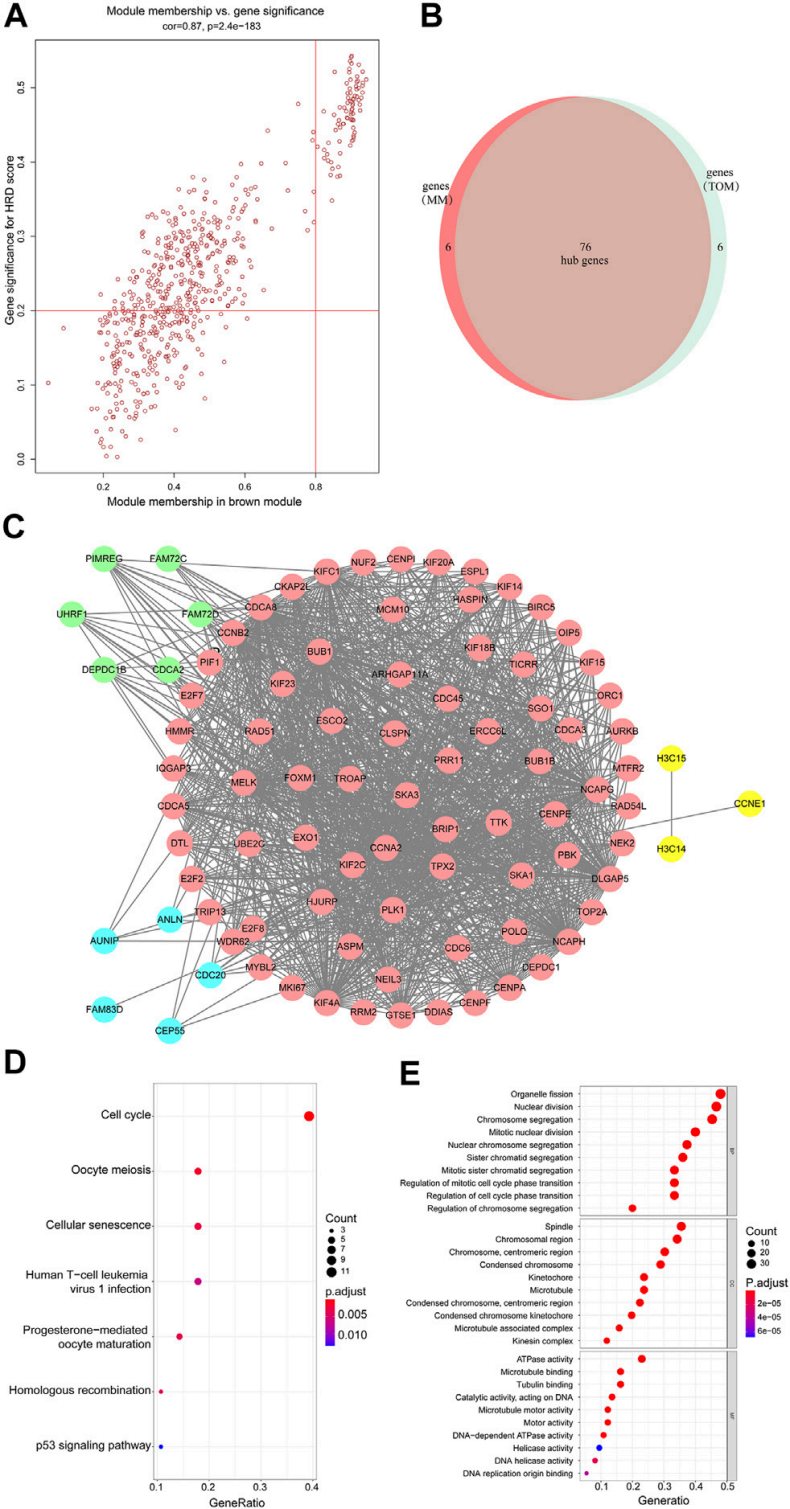
**(A)** Scatterplot of gene significance related to module membership in the brown module. **(B,C)** 76 hub genes were identified. **(D)** KEGG enrichment analysis of the differentially expressed genes shown as a scatterplot. The enrichment degree of KEGG was measured by the number of genes enriched to this pathway. The horizontal coordinate is the gene ratio. The greater the value of the gene ratio, greater will be the degree of enrichment. The vertical coordinate is the pathway term with high enrichment. The size of the dot indicates the number of different genes under this term; larger the dot, higher will be the number of genes. **(E)** GO analysis of predicted genes according to the values in the enrichment score under the themes of biological process (BP), cellular components (CC), and molecular function (MF). Color represents translational values about log10 of q value, and size represents the number of genes enrichment. The horizontal axis represents the proportion of gene enrichment. The GO enrichment analysis classifies genes according to their different functions.

In order to understand the potential reason for the alteration of the HRD score, we performed WGCNA to identify essential modules representing highly correlated genes, contributing to both the HRD component and the combined score. The 9,335 samples with clinical information were clustered by an average linkage method and Pearson’s correlation method. The expression values of 6,386 genes were collected to construct co-expressed gene networks in the sample dendrogram and trait heat map (Figure 2A). In this study, the power of *β* = 6 was selected to ensure a scale-free network (Figure 2B,C) (scale-free *R* ^2^ = 0.96, slop = −1.79). After merging some modules through a cutline of 0.25, a total of 15 modules were identified by the dynamic tree cut method. The clustering dendrogram of genes is shown in Figure 2D. A heat map for the module–trait relationship is shown in Figure 2E,F. The brown module containing 592 genes (R = 0.5322; *p* = 0) was most positively correlated with both the HRD combined score and the component score in the heat map of module–trait relationship. Thus, the brown module was selected as the clinical significant module for further analysis. Scatterplots of GS (*y*-axis) versus MM (*x*-axis) for the brown module is shown in Figure 3A. The scatterplot demonstrated that GS and MM had a very significant correlation, and genes in brown module were highly associated with the HRD score.

### Identification of Hub Genes and Construction of Co-Expression Network

The member analysis led to the identification of a total of 82 hub genes with high connectivity, with an HRD score from the brown module based on the cutoff criteria (|MM| > 0.8 and |GS| > 0.2) (Figure 3A). Meanwhile, through evaluating intramodule connectivity, 82 genes were identified with a connectivity degree ≥5 and the connectivity weight threshold set as 0.45 in the TOM-based co-expression network. Last, after merging the results of the previous two approaches, altogether 90 genes were included. The overlapped 76 genes were finally identified as the hub genes (Figure 3B and Supplementary Material S3). From Figure 3B, 76 hub genes were distributed in pink. Genes in blue were non-overlapped ones from selection of |MM| > 0.8 and |GS| > 0.2 in the first approach. Genes in green were non-overlapped ones from the TOM-based analysis in the second approach. Other connected genes were also displayed in yellow (Figure 3C). The Kyoto Encyclopedia of Genes and Genomes (KEGG) and Gene Ontology (GO) analysis of brown members are shown in Figure 3D,E. The KEGG analysis showed that the hub genes of the brown module were enriched in the cell cycle pathway (Figure 3D). GO terms such as organelle fission, nuclear division, chromosome segregation, mitotic nuclear division, and nuclear chromosome segregation for the biological process (BP), spindle, and chromosomal region for cellular components (CC), and ATPase activity for molecular function (MF) were among the statistically overrepresented terms (Figure 3E). Therefore, these bioinformatics analyses suggested that the HRD score was associated with programmed cell division and proliferation. The HRD score in tumor cells was probably dynamically increasing considering that tumor cells multiplied rapidly and were more frequently in a cell division cycle.

### HRD Score Among Cancers Was a Prognostic Factor

**FIGURE 4:**
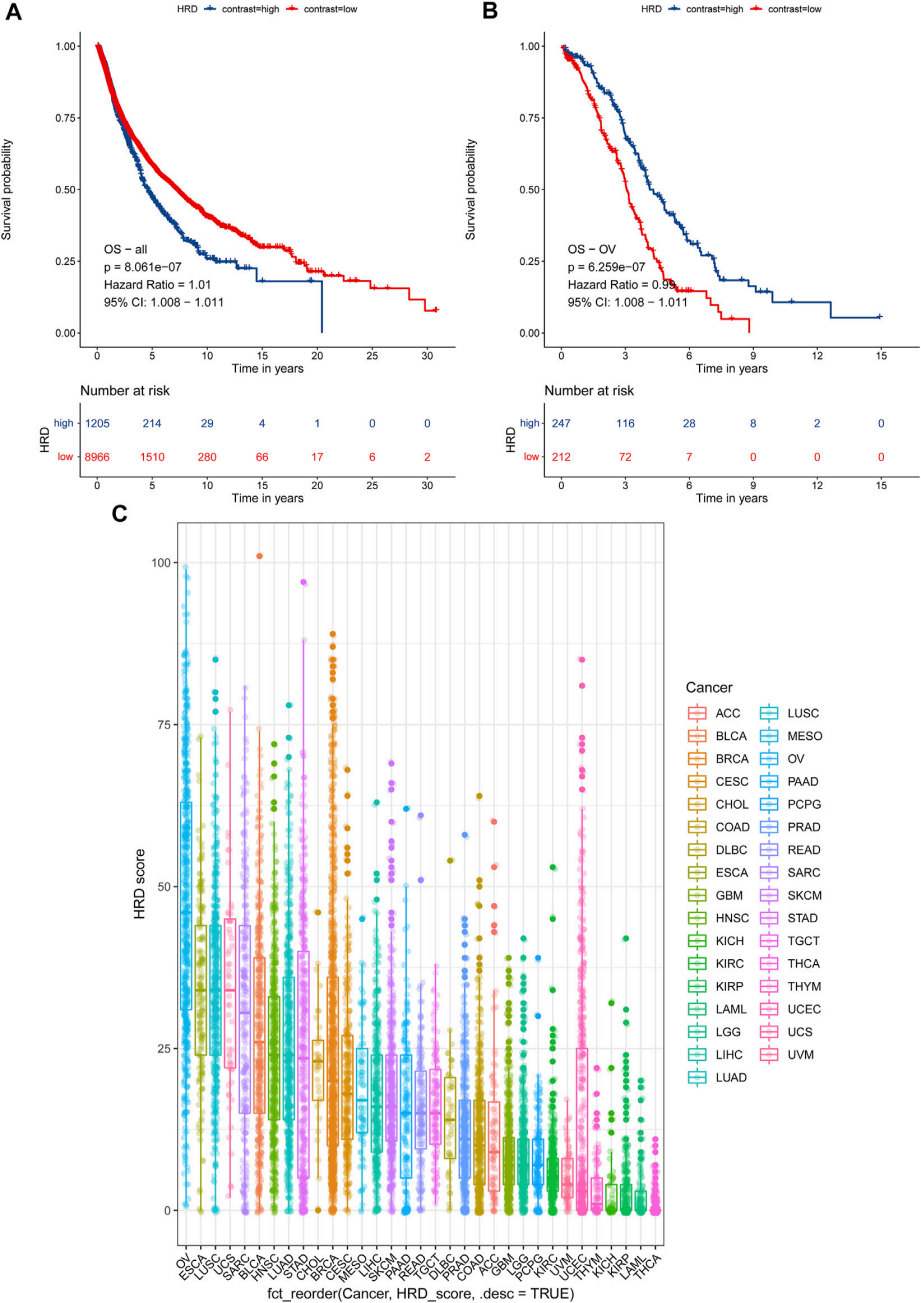
**(A)** Survival analysis of OS between an HRD score ≥42 and an HRD score <42 in 10,171 cases across 33 cancer types, HR = 1.01, 95% CI = 1.008–1.011, *p* < 0.001. **(B)** Survival analysis of OS between an HRD score ≥42 and an HRD score <42 in 459 cases of ovarian cancer, HR = 0.99, 95% CI = 0.98–0.99, *p* < 0.001. **(C)** HRD combined score distribution across 33 cancer types in 10,619 cases.

In light of the cumulative DNA damage manifested by the HRD score in tumor cells, we tried to explore HRD score’s role in predicting patient survival. First, we tested combined HRD score associations with overall survival (OS) across 33 cancer types in 10,171 cases. The combined HRD score ≥42 was associated with shorter OS than the HRD score <42 cohort in altogether 33 cancer types from 10,171 cancer cases in TCGA (HR = 1.010, 95% CI: 1.008–1.011, *p* < 0.001) (Figure 4A). However, as for ovarian cancer, HRD ≥42 cohort was significantly associated with longer OS than the HRD score <42 cohort in 459 HGSC cases (HR = 0.99, 95 % CI: 0.98–0.99, *p* < 0.001) (Figure 4B). Next, we investigated the HRD score distribution among 33 types of cancer and found that ovarian cancer had the top highest HRD combined score and component scores, followed by lung cancer, esophagus cancer, and cervical cancer (Figure 4C, Supplementary Material S4). Ovarian cancer was the only cancer type with the median HRD combined score that outnumbered 42. Thyroid cancer was with the lowest HRD combined and component scores among the 33 cancers.

**FIGURE 5:**
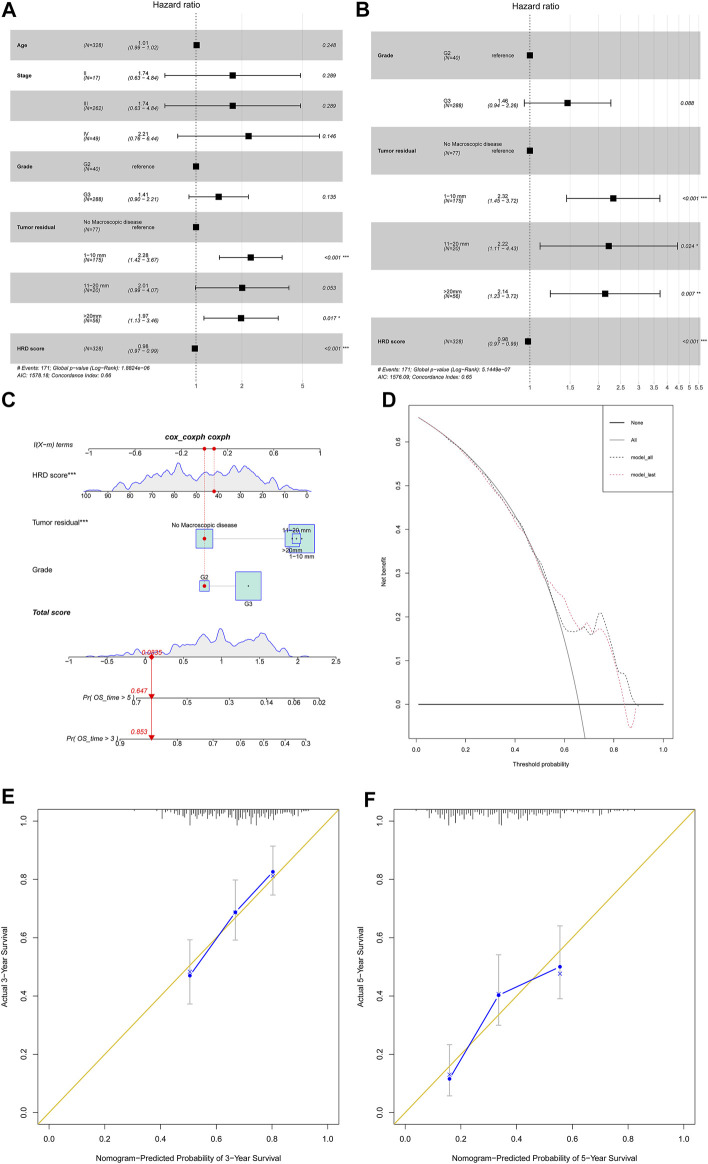
**(A)** Forest plot displayed the association of clinical characteristics with OS by the COX regression analysis, including factors like patient age, tumor stage, tumor grade, tumor residual, and HRD score. **(B)** Forest plot displayed the COX regression analysis for OS with stepwise bidirectional elimination selection. **(C)** Nomogram to predict the 3- and 5-year survival. Pr: probability. **(D,E)** Calibration curve for 3- and 5-year OS nomogram models. The gold line represents the ideal nomogram, and the blue line represents the observed nomogram. **(F)** Decision curve analysis. Net benefit curves for model with three characteristics compared with model with all characteristics. *y*-axis measures net benefit which is calculated by summing the benefits (true positives) and subtracting the harms (false positives).

Last, we tried to investigate the HRD score impact on 328 primary HGSC patient survival outcomes. First, we investigated several clinical variables impact on overall survival by univariate COX regression (Supplementary Material S5). The HRD score, tumor stage, and tumor residual after primary cytoreductive surgery made an impact on the overall survival. Next, we built multivariate COX proportional hazard models after adjusting for variables like patient age, tumor stage, tumor grade, and tumor residual (Figure 5A). We also applied stepwise regression selection with bidirectional elimination in Cox regression, and three variables including tumor grade, tumor residual, HRD scores were selected in the model (Figure 5B). From above, the HRD score was the independent factor that the higher combined HRD score was associated, with better OS in HGSC (HR 0.98, 95% CI 0.97–0.99, *p* < 0.001; HR 0.98, 95% CI 0.97–0.99, *p* < 0.001, respectively). Additionally, smaller tumor residual was statistically associated with better OS.

In order to develop a model predicting odds of OS over 3 and 5 years in HGSC patients, a nomogram (Figure 5C) was generated considering the three prognostic variables including the HRD score, tumor residual, and tumor grade on the basis of the multivariate analysis above (Figure 5B). By calculating the total score, clinicians can stratify patients into distinct risk groups and recommend closer clinical follow-up. From Figure 5, the HRD score at 42 seemed to add little impact on patient survival. The calibration plots for 3-year OS and 5-year OS rates showed an optimal agreement between prediction by the nomogram and the actual observation (Figure 5D,E). The decision curve analysis revealed that these three characteristics predicted OS and all characteristics for a large probability threshold (Figure 5F). According to the prognosis stratification, our model could improve clinical decision making, including HGSC patient postoperative tumor marker monitor, first-line chemotherapy choice, maintenance of treatment implementation, and design of clinical trials.

## Discussion

In this study, we first found that a higher HRD score was linked to higher tumor stages and recurrent tumor tissue demonstrated a slightly higher HRD score than the primary counterpart. We further constructed 15 co-expression modules with the WGCNA method, identified genes impacting the HRD score, and proved that the HRD score was tightly associated with cell division and proliferation. A higher HRD score across 33 cancer types was correlated with worse survival outcomes. Contrary to other cancer types, the HRD score in ovarian cancer ranked the top among 33 cancer types and was positively associated with patient survival. In HGSC, higher HRD score and smaller tumor residual predicted better survival outcomes. Meanwhile, we developed and validated a predicting model for 3- and 5-year overall survival prediction in ovarian cancer patients.

Considering the essential role played by the HR pathway in ensuring tumor cells survival after defective DNA damage, we identified the intriguing relationship between the HRD score and clinical factors by analyzing clinical data containing 33 cancer types. We showed that the HRD score climbed with the rising of tumor stage and indicated a bad prognosis. The recurrent tumor tissue displayed a higher HRD score than the primary one. These associations were compatible in correlating the accumulation of chromosomal defects burden with advanced tumor stage, tumor recurrence, and worse prognosis. The HRD score measured chromosomal abnormality including chromosomal breaks span area >10mb, the absence of paternal or maternal copies, and appearance of allelic imbalance involving telomere. These genomic scars represent a historical record of DNA damage exposure and attempt of alleviating DNA damage by the HR pathway. The disruption of DNA damage repair led to the accumulation of large-scale genome instability over time with the consequence of the increasing HRD score (Jeggo et al., 2016). Cancer progressed from the initiation of malignancy to the advanced stages coupled with the building up of genomic scar. The HRD score appeared to be one-way forward accelerating in tumor progression and metastasis (Stover et al., 2020). The lost chromosomal pieces were barely brought back due to the absence of template and the genomic scars were hard to be healed. Another study also displayed that the HRD score in ovarian cancer with BRCA1/2 mutations tended to be higher in the recurrent than in the primary sample (Patel et al., 2018). However, they did not take into account the samples of BRCA wild-type. From the dot plot they displayed, we could still see a higher HRD score in overall recurrent sample than in the primary sample. We considered that tumor distant metastasis involves more of epithelial mesenchymal transition (EMT) instead of intervened programmatic DNA replication. Hematogenous and lymphatic metastasis might be explained by other mechanisms which involved less of the accumulation of complex genome error over time. This was attributed to the reasons that tumor stage IV was not associated with a higher HRD score. Another research also found no significant genomic variations including driver genes mutation and copy number variations between primary and metastatic sites of HGSC(Lee et al., 2020).

A higher HRD score in ovarian cancer predicted a better prognosis unlike other cancer types. Ovarian cancer was found at the top of the list of the median HRD combined score and was the only cancer type with the HRD score that outnumbered 42. A higher HRD score meant less integrity of genome and rendered cancer cells vulnerable to loss of DNA repair proteins. Like every double-edged sword, ovarian cancer possessed improved sensitivity to DNA-damaging agents, namely, PARPis, by taking advantage of “synthetic lethality.” Synthetic lethality is the inability to tolerate the simultaneous loss of two important functions, literally the integrity of genome and DNA repair proteins, leading to cell death. PARPis will be most active in tumors with a higher HRD score, while tumors without HRD are unlikely to respond to PARPis (Stover et al., 2020). Ovarian cancer possessed improved sensitivity to DNA-damaging agents like platinum and PARPis, which intervened tumor cell division cycle and proliferation, since the accumulation of genomic scar summited in ovarian cancer. HGSC received platinum and PARPis as first-line standard chemotherapy, while cancer types found at the bottom list generally do not receive these as first-line standard therapy. Thus, patients who were higher HRD-scored and subsequently superior sensitive to chemotherapy and PARPis showed better survival. Our results were similar with other studies (Marquard et al., 2015; Knijnenburg et al., 2018; Takaya et al., 2020), while we dig further in the origination and alteration of the HRD score and the mechanisms behind the WGCNA method and built the model of ovarian cancer patient survival prediction with preliminary 328 cases and validated the model in several ways. Another study also showed the median number of rearranged chromosome arms was associated with cancer prognosis (Fearon and Vogelstein, 1990). According to our analysis, the HRD score hold promise an important prognostic factor in epithelial ovarian cancer. Our data strengthened the rationale for extending the clinical use of HRD biomarker’s role in predicting responses to DNA-damaging agents and warranted larger clinical research (Konstantinopoulos et al., 2015).

HGSC with BRCA mutation showed PARPis sensitivity with long treatment free interval (Tan et al., 2008; Foo et al., 2021; Kim et al., 2021). BRCA mutation seemed to be one indicator for PARPi treatment. BRCA1/2 served as a critical modulating factor in the HR pathway by co-localizing with RAD51, interacting with P53, and a set of proteins to format a remodeling complex, and in this way made fundamental impact on HR score evaluation (Lord and Ashworth, 2016). However, gene loss or mutation in the HR pathway other than BRCA1/2, such as RAD51, RAD54, ATM, TP53, MRE11, and XRS2, also brought in the genomic and chromosomal instability and contributed to cell growth arrest impacting the ultimate HRD score (Khanna and Jackson, 2001). Genetic or epigenetic changes in the HR pathway, once recognized, can also be therapeutically targeted through synthetic lethality. Cancers without BRCA1/2 loss but with accumulation of similar genomic footprints displayed increased sensitivity to DNA-damaging agents. Whether the malfunctions of other DNA damage repair like non-homologous end joining (NHEJ) and DNA single-brand break repair (SSB) would make an effect on the HRD score needed to be verified. BRCA mutation condition alone limitedly discriminated the response to DNA-damaging agents. Rapidly proliferating tumor cells and hemopoietic stem cells with a higher HRD score were more easily targeted by DNA-damaging agents than the relatively quiescent somatic cells, regardless of BRCA1/2 expression. Human somatic cells with germline BRCA1/2 mutation were not globally harmed by PARPis. Somatic non-neoplastic and neoplastic cells with germline BRCA mutation in one person demonstrated varied sensitivity to PARPis. Varying degree of anemia was observed clinically due to the different extents of HR deficiency in hemopoietic stem cells among patients. Some ovarian cancer patients with BRCA mutation developed myelodysplastic syndrome and acute myeloid leukemia upon PARPi treatment (Churpek et al., 2016; McNerney et al., 2017; Morice et al., 2021).

The clinical validity of the HRD score in cancer is better evaluated in terms of PARPi benefit (Miller et al., 2020). Several trials have demonstrated that HRD genomic scars predicted different extents of response to DNA-damaging agents in breast cancer (Isakoff et al., 2015; Telli et al., 2016; Loibl et al., 2018; Telli et al., 2018). For ovarian cancer, recently clinical trials were performed with patients being stratified by HRD status, incorporating PARPi maintenance in primary and platinum-sensitive recurrent ovarian cancer settings. An HRD score of ≥42 was determined to signify HRD (HR-deficient), and a score of <42 was considered HR-proficient in clinical trials. In PAOLA-1 and PRIMA clinical trials, HRD tests could discriminate the response to PARPis that HR-deficient populations showed great benefit compared to HR-proficient populations in primary HGSC patients. (González-Martín, 2019; Ray-Coquard et al., 2019 #167 #168) In 2021, the Society of Gynecologic and Oncologists (SGO), PAOLA-1 presentation showed the gene test by next-generation sequencing or gene panel cannot substitute the HRD score. Mutation in genes in the HR pathway did not necessarily change the effectiveness of this pathway and guarantee the PARPis response. PARP inhibition killed tumor cells by coordinating with the considerable accumulation of genomic defects by HR deficiency, rather than the mutated BRCA1/2. That explained why some patients with germline BRCA1/2 mutation showed no response to PARPis. The accumulated genomic scar has not reached the point. “Synthetic lethality” was advantageous only when DNA damage and chromosomal scars were accumulated and the HRD score was summited. We proved the HRD score accelerated as cells divided and proliferated. A continuous HRD score is more informative than binary cutoff. This is attributed to reasons that ARIEL3 and NOVA clinical trials showed platinum-sensitive recurrent HGSC patients could generally benefit from PARPis, regardless of BRCA mutation or any gene test result (Coleman et al., 2017; Del Campo et al., 2019), since the DNA damage ascended in this recurrent population. Both FDA and NCCN guidelines 2020 approved and recommended PARPi treatment in platinum-sensitive recurrent HGSC without the mandatory gene test results. The quantitative HRD score per se acted as an important predictive biomarker in personalizing the use of PARPis and platinum-based chemotherapy and patient prognosis.

Notably, archival tissue, namely, primary tumor tissue, was extracted and tested for the HRD score in AREIL3 and NOVA, instead of the pretreatment tissue, namely, recurrent tumor tissue. The median time between primary and recurrent biopsy was 2.7 years (Haunschild and Tewari, 2021). Genomic testing on initial surgery tissues in these trails did not represent the real-time genomic and chromosomal instability situation at recurrence. The ongoing proliferating tumor cells with heterogeneity and apparent adaptability under selective pressure by chemotherapy likely changed the genome scars and accumulated more genome damage and instability. A real-time sophisticated HRD assay performed on a biopsy of the recurrent tumor is able to capture tumor evolution processes and track the alteration of the HR function in response to therapy-selective pressure (Pellegrino et al., 2019). Studies comparing HRD scores between paired primary HGSC tissue and recurrent counterpart, and between neoplastic tissue and non-neoplastic tissue from the same patient, may better explain this.

## Conclusion

In summary, we first discovered that the higher HRD score was linked to higher tumor stages, and recurrent tumor tissue demonstrated slightly higher HRD score than the primary counterpart. We demonstrated that the HRD score representing the accumulated genomic scars was dynamically increasing in proliferating tumor cells, since the HRD score was tightly correlated to tumor cell division and replication from bioinformatics analysis. We highlighted quantitative HRD score biomarker’s role in predicting therapeutic response to DNA-damaging agents and patient survival outcomes in subsets of cancers including ovarian cancer.

## Data Availability

The original contributions presented in the study are included in the article/Supplementary Material; further inquiries can be directed to the corresponding authors.
