# Supplementary material for: Dynamically Accumulating Homologous Recombination Deficiency Score Served as an Important Prognosis Factor in High-Grade Serous Ovarian Cancer
Source: Front Mol Biosci. 2021 Nov 19;8:762741. doi: 10.3389/fmolb.2021.762741 (PMC8640082; doi:10.3389/fmolb.2021.762741)
Supplement: Supplementary file 3 [file Table5.DOCX]

**Supplementary File 5 : Univariate COX regression of clinical factors impact on overall survival**

|  | Hazard Ratio (95%CI) | P |
| --- | --- | --- |
| Age (year)(N=328) | 1.013(0.999-1.028） | 0.072 |
| HRD score(N=328) | 0.983(0.975-0.990) | <0.001 |
| Tumor grade |  |  |
| G2(N=40) | Reference |  |
| G3(N=288) | 1.395(0.903-2.153) | 0.133 |
| Tumor stage |  |  |
| II(N=17) | Reference |  |
| III(N=262) | 3.012(1.113-8.151) | 0.030 |
| IV(N=49) | 3.378(1.184-9.642) | 0.023 |
| Tumor residual |  |  |
| No macroscopic disease | Reference |  |
| 1-10mm(N=175) | 2.283(1.427-3.652) | <0.001 |
| 10-20mm(N=20) | 2.175(1.088-4.350) | 0.028 |
| >20mm(N=56) | 1.952(1.124-3.387) | 0.017 |
|  |  |  |
